# Community barcoding reveals little effect of ocean acidification on the composition of coastal plankton communities: Evidence from a long-term mesocosm study in the Gullmar Fjord, Skagerrak

**DOI:** 10.1371/journal.pone.0175808

**Published:** 2017-04-26

**Authors:** Julia A. F. Langer, Rahul Sharma, Susanne I. Schmidt, Sebastian Bahrdt, Henriette G. Horn, María Algueró-Muñiz, Bora Nam, Eric P. Achterberg, Ulf Riebesell, Maarten Boersma, Marco Thines, Klaus Schwenk

**Affiliations:** 1 Alfred-Wegener-Institut Helmholtz-Zentrum für Polar- und Meeresforschung, Biologische Anstalt Helgoland, Germany; 2 Biodiversity and Climate Research Centre (BiK-F), Senckenberg Gesellschaft für Naturkunde, Frankfurt am Main, Germany; 3 Institute of Ecology, Evolution and Diversity, Faculty of Biological Sciences, Frankfurt am Main, Germany; 4 University Koblenz-Landau, Institute of Environmental Science, Landau in der Pfalz, Germany; 5 GEOMAR Helmholtz Centre for Ocean Research Kiel, Kiel, Germany; 6 University of Bremen, Bremen, Germany; CSIR-National Institute of Oceanography, INDIA

## Abstract

The acidification of the oceans could potentially alter marine plankton communities with consequences for ecosystem functioning. While several studies have investigated effects of ocean acidification on communities using traditional methods, few have used genetic analyses. Here, we use community barcoding to assess the impact of ocean acidification on the composition of a coastal plankton community in a large scale, *in situ*, long-term mesocosm experiment. High-throughput sequencing resulted in the identification of a wide range of planktonic taxa (Alveolata, Cryptophyta, Haptophyceae, Fungi, Metazoa, Hydrozoa, Rhizaria, Straminipila, Chlorophyta). Analyses based on predicted operational taxonomical units as well as taxonomical compositions revealed no differences between communities in high CO_2_ mesocosms (~ 760 μatm) and those exposed to present-day CO_2_ conditions. Observed shifts in the planktonic community composition were mainly related to seasonal changes in temperature and nutrients. Furthermore, based on our investigations, the elevated CO_2_ did not affect the intraspecific diversity of the most common mesozooplankter, the calanoid copepod *Pseudocalanus acuspes*. Nevertheless, accompanying studies found temporary effects attributed to a raise in CO_2_. Differences in taxa composition between the CO_2_ treatments could, however, only be observed in a specific period of the experiment. Based on our genetic investigations, no compositional long-term shifts of the plankton communities exposed to elevated CO_2_ conditions were observed. Thus, we conclude that the compositions of planktonic communities, especially those in coastal areas, remain rather unaffected by increased CO_2_.

## Introduction

Enhanced atmospheric CO_2_ concentrations result in an increase in surface ocean *p*CO_2_ with a subsequent decrease in pH. Several studies have investigated the effects of ocean acidification (OA) on marine biota under laboratory and field conditions. Generally, OA has been shown to negatively affect survival, calcification, growth and reproduction of a range of organisms [1]. However, there is significant variation among marine species in their sensitivity to elevated CO_2_. Direct and indirect effects of OA on single species, which retard development [2], reduce reproduction [3, 4] and survival [5], may also alter community structures [6] and thereby impact the food web [7, 8]. Nevertheless, investigations during large-scale mesocosm field studies in Espegrend (Bergen, Norway) and Kongsfjorden (Ny-Ålesund, Svalbard) showed an overall resilience of plankton communities (bacteria, phytoplankton, micro- and mesozooplankton) towards enhanced CO_2_ concentrations, whereby observed biological responses were largely depending on temperature and nutrient availability [9–13].

Up to now possible changes in the community composition of planktonic organisms were mainly based on optical (microscopic and flow cytometry) investigations, where the actual diversity of species may be underestimated, especially in organism groups with small morphological differences. Marine planktonic taxa (phyto-, bacterio- and zooplankton) are highly diverse and the differentiation at genus and species level is time consuming and requires highly specialized taxonomic expertise. Additionally, there is a potentially high diversity of cryptic species as well as larval stages, which are not captured by morphological investigations. New molecular techniques show rapid advancements, with promising tools for species identification. Especially the combination of DNA barcoding and next generation sequencing (community barcoding) can provide accurate and high-resolution taxonomic data [14–17].

In the long-term mesocosm experiment in Kristineberg (west coast of Sweden), which was part of the BIOACID II project, the response of different trophic levels to elevated CO_2_ concentrations during a winter-to-summer plankton succession over 113 days was investigated (also see other publications within this collection). Within that framework our present study focused on the assessment of the diversity of a broad spectrum of the biocoenosis including all trophic levels (producers, consumers, decomposers).

DNA barcoding in combination with high-throughput sequencing (HTS) was utilized to investigate potential CO_2_ induced shifts in the plankton community compositions. We hypothesized that OA may induce taxonomic shifts in the planktonic community composition, which are potentially undetectable using morphological techniques, but might be revealed using genetic methods.

## Methods

### Experimental design

**Fig 1:**
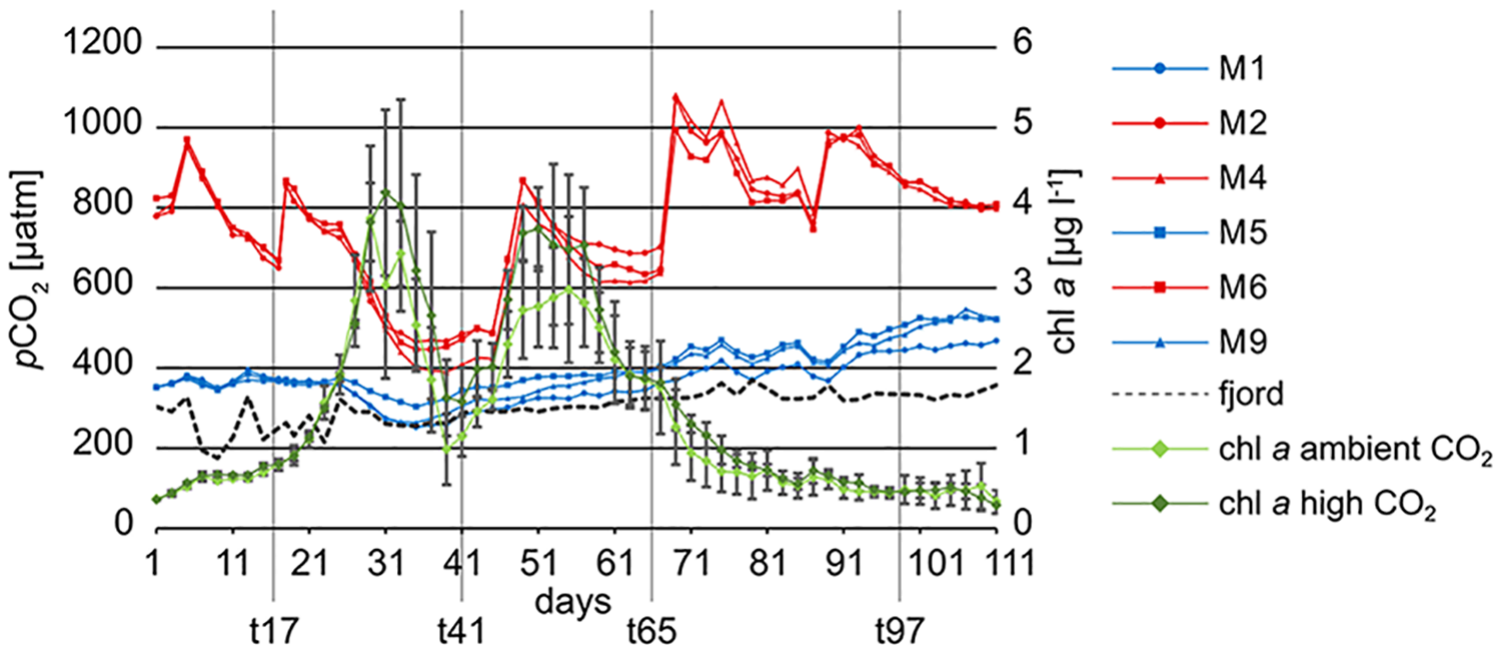
*p*CO_2_ and chlorophyll *a* concentration in mesocosms over the experimental period. High and ambient CO_2_ mesocosms are represented by red and blue lines, respectively. The fjord is indicated by a black dashed line. The mean chlorophyll *a* concentration in the selected high and ambient CO_2_ mesocosms is represented by a dark and light green line, respectively. X axis: experimental days. Left y axis: *p*CO_2_ concentrations. Right y axis: mean chlorophyll *a* concentrations. Data pooled from the three ambient and three high CO_2_ mesocosms, that were also used for the community barcoding. Error bars = SD.

In Gullmar Fjord, Sweden (58° 15’ 9 N, 11° 28’ 7 E) ten pelagic mesocosms reaching to 19 m water depth were deployed. All of them enclosed roughly 50 m^3^ of seawater and contained all organisms present in the fjord which were < 1 mm at the time the mesocosms were closed (note, however, that some larger species were included in the study later on). The field location was not privately owned or protected, and neither endangered nor protected species were involved. No specific permission was required for activities related to field sampling. Organisms grew under *in situ* temperature conditions and close to *in situ* light levels. Five of the mesocosms were manipulated with CO_2_ enriched water (~ 760 μatm), the others remained unchanged to serve as controls (~ 380 μatm). Due to net outgassing, the CO_2_ concentration decreased in the high CO_2_ mesocosms and needed to be re-adjusted repeatedly. However, the ambient and high CO_2_ treatment remained different throughout the experiment (Fig 1). As some taxa appearing later in the seasonal succession might not have been present in the water column when the mesocosms were closed, we added 22 L of water from the fjord every fourth day to each mesocosm. Additional to smaller plankton, we also added herring eggs (*Clupea harengus*) and green sea-urchin gastrula stages (*Strongylocentrotus droebrachiensis*) to the mesocosms. Those species were not present in the fjord when the mesocosms were closed. Adult herring, caught in the Oslo fjord were obtained from a local fisherman. In the laboratory eggs from the dead herrings were stripped off and fertilized. Afterwards, eggs were stuck on plastic plates and placed in the middle of each mesocosm at 3 m depth from day 48 until peak hatching on day 63. Around day 71 after they reached the yolk-sac stage herring larvae presumably fed on copepod nauplii and ciliates. With growing size, they switched to larger prey. This side experiment was performed under the ethical permission number 332–2012 (issued by the Swedish Board of Agriculture "Jordbruksverket"). The species used is not endangered and animal welfare was assured by minimization of stress from handling and treatment. The CO_2_ concentrations in the mesocosms was far below the lethal level and sacrificed specimens were anaesthetized in advance with MS-222, to reduce stress to a minimum. Green sea-urchin larvae were cultured in the laboratory following Dorey et al. [18]. When they reached the swimming gastrula stage they were gently added to each mesocosm on day 56 (for further details see Dupont et al., this collection).

CTD (Conductivity-Temperature-Depth) casts were taken every second day between 11 am and 3 pm to monitor *inter alia* the development of salinity, temperature and pH in the mesocosms and fjord. Depth-integrated water samples were taken every second day using an Integrating Water Sampler (IWS, Hydro-Bios, Kiel, Germany) which sampled a total volume of 5 L evenly distributed over the depth range from 0–18 m. From this water among other properties, concentrations of nitrate (NO_3_^-^), nitrite (NO_2_^-^), dissolved silicate (SiO_4_^3-^), ammonium (NH_4_^+^), and phosphate (PO_4_^3-^) were measured using standard auto-analyser (Seal Analytical QuAAtro) techniques according to Murphy and Riley (1962) [19], Hansen and Grasshoff (1983) [20] and Holmes et al. (1999) [21], in addition to nanomolar nutrient techniques [22] for periods with depleted nutrient levels. In addition, pigment analyses [23] and microzooplankton microscopy [24] were performed. Mesozooplankton samples were taken with an Apstein net (55 μm mesh size, 1 m long, Hydro-Bios) from 17 m depth to the top of each mesocosm (Algueró-Muñiz et al. 2017, this collection). The chlorophyll *a* concentrations measured via pigment analysis indicated two bloom events which occurred simultaneously in the ten mesocosms (Fig 1). Further details on mesocosm setup and performance as well as CO_2_ manipulation can be found in the overview paper of the PLoS collection [23].

Plankton samples were taken every 8^th^ day from each mesocosm plus the surrounding fjord. For the mesoplankton (> 200 μm) samples the content of the mesozooplankton net hauls was pre-screened with a 200 μm sieve and fixed with 90% pure ethanol in 100 mL bottles. To sample small-sized plankton including pico-, nano- and microplankton (> 0.45 μm and < 200 μm), 500 mL of water sampled with the IWS was sieved with a 200 μm mesh to remove large phyto- and zooplankton. The sieved water was subsequently filtered onto a nylon filter (0.45 μm pore size, 25 mm diameter, Whatman^®^) and fixed with 99.9% pure ethanol in 2 mL tubes. For the community barcoding, filter and net samples from three randomly selected ambient (M1, M5, M9) and high (M2, M4, M6) CO_2_ mesocosms, and the fjord, from four time points (t17, t41, t65, t97), were used (in total 28 samples). In this study, we focused on long-term effects of OA on the composition of plankton communities, as the number of samples for the HTS was financially limited. Therefore, we selected the time points to be equally distributed over the experimental period, before and after the bloom events.

### DNA isolation

For DNA isolation we used a modified version of a phenol/chloroform method [25]. To extract the DNA of the mesoplankton the sample bottles were gently homogenized, 2 mL sample were transferred into a 2 mL tube and ethanol was removed with a pipette. This procedure was repeated until 0.25 mL (maximum number of organisms per isolation tube) of the tube was filled with organisms. In total, 10% (10 mL) of each mesoplankton net sample was used for DNA isolation. Samples were dried at 40°C to completely remove the ethanol. Nylon filter carrying pico-, nano- and microplankton were placed in a petri dish, cut in small pieces and dried at 40°C. The filter pieces were put in 2 mL tubes with three metal beads (3 mm) and frozen overnight at -80°C. The frozen filter pieces were tissue-lysed once for 50 s at a frequency of 20 Hz (TissueLyser II, Qiagen) to crush phytoplankton cells. For cell lysis 800 μL lyse buffer (50 mM Tris-HCl pH 8, 200 mM NaCl, 0.2 mM EDTA, 0.5% SDS, 0.1 mg mL^-1^ proteinase K, 0.25 mg mL^-1^ glycogen) was added. Subsequently samples were vortexed, centrifuged and incubated at 37°C overnight with gentle mixing. Then 4 μL RNase (100 mg mL^-1^) was added followed by another incubation at 37°C for 15 min. The buffer and RNase volume was doubled for the mesoplankton samples. Afterwards the lysate was transferred into new 2 mL tubes whereby the mesoplankton solution was divided into two tubes. Filter and organism leftovers were not transferred. Then 804 μL phenol/chloroform/isoamyl alcohol (25:24:1) were added. These samples were vortexed and centrifuged at 13000 g for two min. Subsequently the liquid supernatant was pipetted into a new 2 mL tube followed by another addition of 4 μL RNase and an incubation at 37°C for 15 min with gentle mixing. Then the phenol/chloroform addition was repeated and the liquid supernatant was again transferred into new 2 mL tubes (maximum 400 μL). Afterwards, 36 μL sodium acetate solution (3 M) and 900 μL pure ethanol (98%) were added, the samples were vortexed and centrifuged at 8000 g for 10 min. The liquid above the DNA pellets was removed and pellets from the same sample were pooled and washed twice with 500 μL pure ethanol (70%). Then the pellets were dried at 40°C and resuspended in 100 μL 1x TE buffer (10 mM Tris, 1 mM EDTA). Until further use the isolated DNA was stored at -20°C.

### Polymerase Chain Reaction (PCR)

We analysed a part (V4 and V5) of the small subunit rDNA (18S) to cover a potentially wide range of marine planktonic taxa in the community barcoding [26]. Furthermore, we sequenced the more variable gene region cytochrome-c-oxidase subunit I (*cox*1) to achieve a sufficient resolution for species identification. The gene regions 18S and *cox*1 were amplified using the primer pairs 554f (5`-AAGTCTGGTGCCAGCAGCCGC-3`) / 1282r (5`-TCACTCCACCAACTAAGAAGGGC-3`) and LCO1490_t1 (5`- TGTAAAACGACGGCCAGTGGTCAACAAATCATAAAGA-3`) / HCO2198_t1 (5`- CAGGAAACAGCTATGACTAAACTTCAGGGTGACCAAA-3`), respectively [27–29]. Amplification was carried out in a total volume of 20 μL (*cox*1) and 25 μL (18S) using ~ 10 ng DNA. The reaction mix contained 2.5 (*cox*1), 1.25 (18S), mM MgCl_2_, 1 x PCR-buffer, 0.2 (*cox*1), 0.25 (18S) mM dNTPs, 0.2 (*cox*1), 0.3 (18S), μM each primer and 0.5 (*cox*1), 0.1 (18S) units TAQ polymerase. Additionally, 0.1 μg μL^-1^ BSA were added for the 18S PCRs. PCR conditions for *cox*1 were 2 min at 95°C, followed by 35 cycles of 1 min at 95°C, 1 min at 45°C, and 1 min 30 s at 72°C, and a final elongation for 7 min at 72°C. For 18S, the PCR conditions were 2 min at 95°C, followed by 34 cycles of 30 s at 95°C, 1 min at 56°C, and 1 min 30 s at 72°C, and a final elongation for 10 min at 72°C. PCR-products were purified using the ISOLATEII PCR and Gel Kit from Bioline. Clean PCR-products were used as templates for a barcode PCR in which the DNA fragments of each sample were tagged with a specific barcode to enable the identification after the multiplexed sequencing. Therefore, primers were labeled with tags of 6–7 bps. Amplifications were carried out in a total volume of 25 μL using ca. 10 ng PCR product as template. The reaction mix contained 1 x Phusion^®^ High Fidelity buffer, 0.2 mM dNTP’s, 1 μM each primer, and 0.02 units Phusion^®^ High Fidelity polymerase. PCR conditions for *cox*1 were 2 min at 98°C, followed by 27 cycles of 20 s at 98°C, 30 s at 45°C, and 1 min at 72°C, and a final elongation for 6 min at 72°C. For 18S the PCR conditions did not change—only the number of cycles was reduced to 27. Amplification success was 99% and was controlled with gel electrophoresis. PCR of the isolated DNA from the net sample of mesocosm six (t97) yielded no amplicon for the *cox*1 gene region.

After purification, 68.57 and 76.80 ng DNA sample^-1^ from the 18S and *cox*1 PCR products were pooled, respectively. The amplicon pool was sequenced on an Illumina MiSeq platform using the paired end (2 x 300 bp) option at Eurofins Genomics (Ebersberg, Germany). Sequence reads are deposited in the European Nucleotide Archive (http://www.ebi.ac.uk/ena/data/view/PRJEB15126).

For a distinct species identification of *Pseudocalanus* spp., which was the most abundant mesozooplankton organism in the mesocosms, it was necessary to generate additional 18S and *cox*1 reference sequences by standard Sanger sequencing of single individuals. PCR conditions and primer were equal to those described above. The sequences are deposited in the PANGAEA data repository (doi:10.1594/PANGAEA.864598).

### Illumina data processing

The Illumina adapter and primer sequences of the raw sequence pairs were trimmed by using the Trimmomatic [30] software. Quality filtration was performed using a window size of 5 bp with an average phred quality score of 25 and a read length cutoff of 220 bp. Furthermore, sequences including ambiguous bases (Ns) were deleted and sequences were again filtered by keeping a minimum quality phred score of 3 per base using the FastQFS tool [31]. Afterwards, based on the barcode sequence, each read was renamed after the corresponding sample ID and related forward and reverse reads were concatenated. Then the barcode and primer sequences were trimmed and all reads were oriented in 5`- 3`direction. The prediction of operational taxonomical units (OTUs) was carried out with the USEARCH v7 software [32]. Thereby both a *de-novo* and a reference-based (reference sequences for each primer i.e., 18S and *cox1* were downloaded from the NCBI database) chimera filters were applied. The minimum cluster size was three reads with an identity cutoff of 99%. To further de-multiplex sequence data, the 18S and *cox*1 reads were re-assembled with a minimum identity of 99% using the program Geneious^®^ version 7.0.4 [33]. Consensus sequences of clustered OTU sequences were generated on the strength of base majority. Afterwards, sequences of new and not further assembled OTUs were aligned and trimmed to the same length. Sequences related to amplification artifacts were manually detected based on their lack of homology to the target regions and deleted from the set. The resulting set of high-confidence OTUs were mapped by the processed reads, only those OTUs were kept which had a mapping support of at least three reads. This filtered set of OTUs was then used for the community analysis and abundance tables were generated keeping only those OTUs which occurred in a minimum of 2 samples (S1 (18S) and S2 (*cox*1) Tables). OTU distribution among net and filter samples were calculated in Microsoft Excel 2016. For species identification OTU representative sequences were aligned with the NCBI nucleotide database (nt) using the BLASTn [34] algorithm. For this, OTUs were separated according to their sample ID. Those from the same time point and CO_2_ treatment were processed together. Further taxonomical classification of the OTUs was carried out by using the MEGAN program [35]. Thereby, we kept only those taxa for which the pairwise identity between the forward and/or reverse part of the assigned OTU sequences and the NCBI reference sequence was 100%. If an OTU assigned to different taxa with equal pairwise identities, we used the next higher taxonomical level. Resulting taxa and their number of assigned OTUs were put together in a table for further community composition analyses (Generated OTUs and the corresponding sequences are deposited in the PANGAEA data repository (doi:10.1594/PANGAEA.864598)).

### Statistics

Data analyses were performed using the *vegan* package [36] in R software (Version 0.99.879, RStudio, Inc.). For the statistical analyses we used two Hellinger transformed [37] OTU datasets of the 18S gene region. The first data set consisted of the raw OTU abundance data, i.e. the number of sequence reads from the HTS. The second data set were the presence-absence-transformed OTU abundance data (as suggested by Borcard, Gillet [38]). In contrast to the presence-absence-transformed data which only reflects the OTU composition, the number of HTS reads per OTU provide additional information on the relative abundance of species, since the amount of sequence reads is positively correlated with the available amount of genetic material. Although the amount of sequence reads per OTU does not reflect real biomasses or abundances of the planktonic taxa on-site, these values can serve as a proxy for relative abundances or biomasses of taxa and can be compared between mesocosms and/or time points. The data set consisting of raw sequence reads per OTU is referred to as “sequence read” data subsequently. Statistical analyses were performed with environmental data which were *log*10(*x* + 1) transformed, to approximate normality, and standardized to bring all of the variables into proportion with one another (*decostand* with the option “*standardize”*). For all statistical analyses, a probability value of *p* < 0.05 was considered significant. To test for significant differences in the OTU composition and/or in the amount of assigned HTS reads per OTU between the size fractions (filter and net samples), permutational multivariate analyses of variance (*adonis*) were performed, based on the Bray-Curtis distance matrix of the 18S presence-absence-transformed and sequence read data. To investigate whether there were significant differences in the OTU composition of the plankton samples between the ambient and high CO_2_ treatment over the experimental period we performed an *adonis*, based on Bray-Curtis dissimilarity matrices of the 18S presence-absence-transformed and sequence read data. OTU composition changes within and between mesocosms and fjord system, were visualized in nonmetric multidimensional scaling (nMDS) ordinations.

The development of the environmental conditions over the experimental period was investigated with a principal component analysis (PCA). For the PCA we considered temperature, salinity and chlorophyll *a* as measured via pigment analysis using HPLC, as well as the concentrations of different nutrients (NO_3_^-^ / NO_2_^-^, PO_4_^3-^, SiO_4_^3-^, NH_4_^+^) and the abundances of micro- and mesozooplankton. Here, these data were only included as covariates, detailed interpretations and discussions are presented elsewhere [23]. In this study, counts of the most abundant microzooplankton group (ciliates) and the most abundant mesozooplankton species (all stages of *Pseudocalanus* spp. (later identified as *P*. *acuspes*)) were considered in the corresponding taxa abundance variables. Chlorophyll *a* measurement in the fjord for day 41 was missing, therefore this data point was extrapolated by averaging the measurements from days 33, 35, 37, 39, 43, and 45. Due to a deviating sampling schedule, ciliate counts from day 103 instead of day 97 were used. Furthermore, biomass calculations of the microzooplankton, which included also different dinoflagellate species, were included in the PCA analysis.

Since absence represents a statistical information in presence-absence-transformed data sets, redundancy analyses (RDA) is preferred over canonical correspondence analysis (CCA) [39]. Additionally, for short gradients as found in the present study, where species abundance or frequency is a linear function, CCA is an inappropriate model [40]. Therefore, OTU compositions of the plankton (pico-, nano-, micro- and mesoplankton) in the mesocosms in dependence of environmental variables were investigated via RDA [41] on the Hellinger-transformed sequence read and presence-absence-transformed data. The stepwise model builder based on permutation tests (*ordistep*) was used to determine the set of significant explanatory environmental variables for the final RDA.

As already mentioned for the sequence read data, the number of assigned OTUs to certain taxa, identified by the BLAST searches and MEGAN analyses, do also not necessarily reflect species abundances and/or biomasses on-site. However, they can be used to test whether the composition of the planktonic taxa was different between the CO_2_ treatments and/or over the time. Therefore, we performed an *adonis* based on the number of assigned OTUs to certain taxa. To investigate potential differences in the diversity of the detected taxa between the high and ambient CO_2_ treatment and/or over the experimental period, we calculated taxonomic diversity indices $(H´\;=\;-\sum_{i=l}^{R}\left(p_{i}\text{ln}p_{i})*\left(-1\right)\right)$ [42], where *p*_*i*_ is the proportion of OTUs belonging to the *i*^th^ taxa in the subset. Furthermore, OTU evenness $\left(\;J`\;=\;\frac{H´}{\text{ln}\left(S\right)}\right)$ [43] (S = total number of detected taxa in the dataset), was calculated to detect imbalances in the number of assigned OTUs among taxa.

We additionally investigated whether elevated CO_2_ affected the intraspecific genetic diversity of *Pseudocalanus* spp. (later identified as *P*. *acuspes*), which was the most abundant copepod throughout the experiment. Therefore, we performed an *adonis* based on presence-absence-transformed and sequence read data, which exclusively included OTUs from this species.

## Results

### Illumina data processing

The Illumina MiSeq sequencing resulted in about five million paired-end sequence reads. The raw reads were processed for Illumina adapter and primer sequences, and filtered in terms of their sequencing quality and sequenced read length. In total, 32.6% of the reads passed the data processing and filtering steps. This filtered set of reads was then further processed to assign sample information (18S and *cox*1) based on their unique oligomer and primer sequence combinations. After categorizing the filtered reads into samples, the oligomer and primer sequences were clipped off, and all sequences were oriented in 5’ to 3’ direction. Using this sample assignment step 653,789 and 3,417 sequences were assigned as 18S and *cox*1 sequences, respectively. Assigned sequences were then clustered into OTUs, which resulted in 7,734 and 96 OTUs for 18S and *cox*1 samples, respectively. After the re-assembly, alignment clean up and mapping, the final 18S and *cox*1 abundance tables contained a high confident set of 740 (S1 Table) and 31 (S2 Table) OTUs, respectively. Steps of the bioinformatics pipeline, including the programs used, and the numbers of obtained sequence reads and OTUs, can be found in the supplementary data (S3 Table). M13 tailed *cox*1 primers were already successfully used for barcoding approaches of e.g. marine crustaceans [44], fish [45] and various freshwater invertebrates [46]. However, in the present study, the use of M13 tailed *cox*1 primers for HTS resulted in an insufficient number of sequence reads. Thus, further analyses were based on the 18S OTU abundance table only. 18S OTUs originated mainly (40.8 ± 3.3%) from filter samples representing the small size fraction including pico-, nano- and microplankton. Only 11.2 ± 1.2% corresponded to the mesoplankton size fraction of the net samples. Furthermore, there was a substantial overlap (47.9 ± 2.1%) between both fractions.

### Community composition and succession (OTUs)

**Fig 2:**
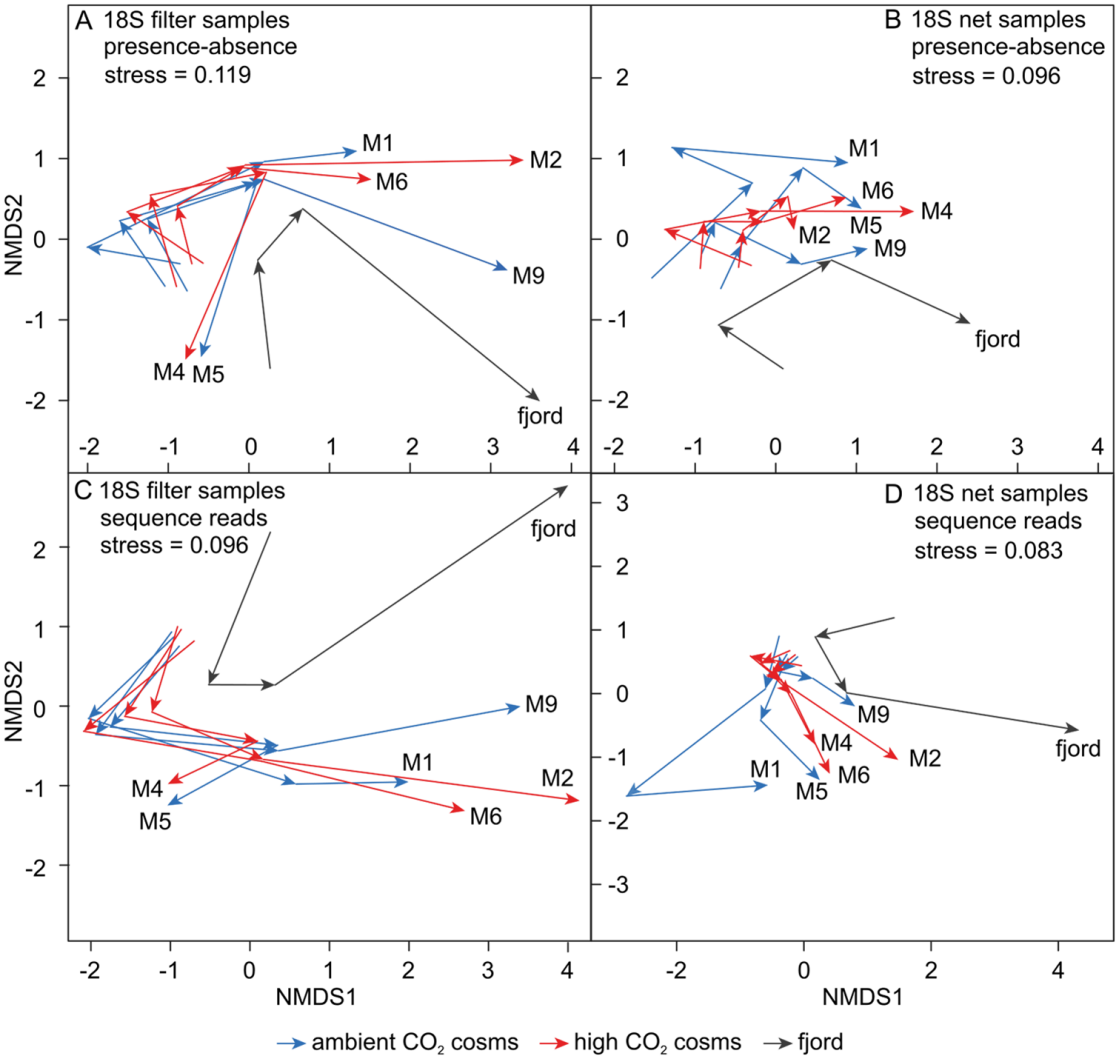
nMDS of OTU compositions over the experimental period. Analysis of (A) filter samples, based on the 18S presence-absence-transformed data, (B) net samples, based on the 18S presence-absence-transformed data, (C) filter samples, based on the 18S sequence read data and (D) net samples, based on the 18S sequence read data. Mesocosms are indicated by their number (M1, M2, M4, M5, M6, M9). In each subplot, the beginning of the first arrow for each mesocosm represents the first time point (t17). The second (t41), third (t65) and fourth (t91) time points are represented by the subsequent arrowheads, respectively. Ambient and high CO_2_ mesocosms are indicated by blue and red arrows, respectively. The fjord is indicated by grey arrows.

The overall OTU composition was significantly different between the size fractions for the 18S OTU presence-absence-transformed and sequence read matrices, respectively, although ~ 50% of the OTUs were present in both filter and net samples (*adonis*: R^2^_0/1_ = 0.30, *p*_0/1_ < 0.05; R^2^_reads_ = 0.21, *p*_reads_ = 0.01). Thus, filter and net samples were analysed separately in the following. Analyses based on the 18S OTU abundance tables revealed no significant differences in the OTU compositions between the ambient and high CO_2_ treatments neither for the presence-absence-transformed (R^2^_filter_0/1_ = 0.01, *p*_filter_0/1_ = 0.96; R^2^_net_0/1_ = 0.04, *p*_net_0/1_ = 0.27) nor for the sequence read data (R^2^_filter_reads_ = 0.012, *p*_filter_reads_ = 0.924; R^2^_net_reads_ = 0.054; *p*_net_reads_ = 0.091), but a significant change occurred over time (R^2^_filter_0/1_ = 0.29, R^2^_net_0/1_ = 0.28; R^2^_filter_reads_ = 0.309, R^2^_net_reads_ = 0.272; *p* < 0.001). Especially at the first and second time point the mesocosms exhibited similar OTU compositions which can be seen also in the nMDS ordinations where they are situated close to each other (Fig 2). With progressing time, distances among mesocosms within the ordination increased, revealing differences in the OTU compositions between the enclosures at the end of the experiment. In the nMDS ordination mesocosms were however not separated by their CO_2_ treatment. The amount of sequence reads per OTU and the OTU composition of the 18S mesoplankton size fraction was significantly different between the mesocosm and fjord samples over the experimental period (R^2^_net_0/1_ = 0.11; R^2^_net_reads;_ *p* < 0.05). However, no such differences were observed for the smaller plankton groups from the filter samples (R^2^_filter_0/1_ = 0.08, *p* = 0.06; R^2^_filter_reads_ = 0.079, *p*_filter_reads_ = 0.09).

**Fig 3:**
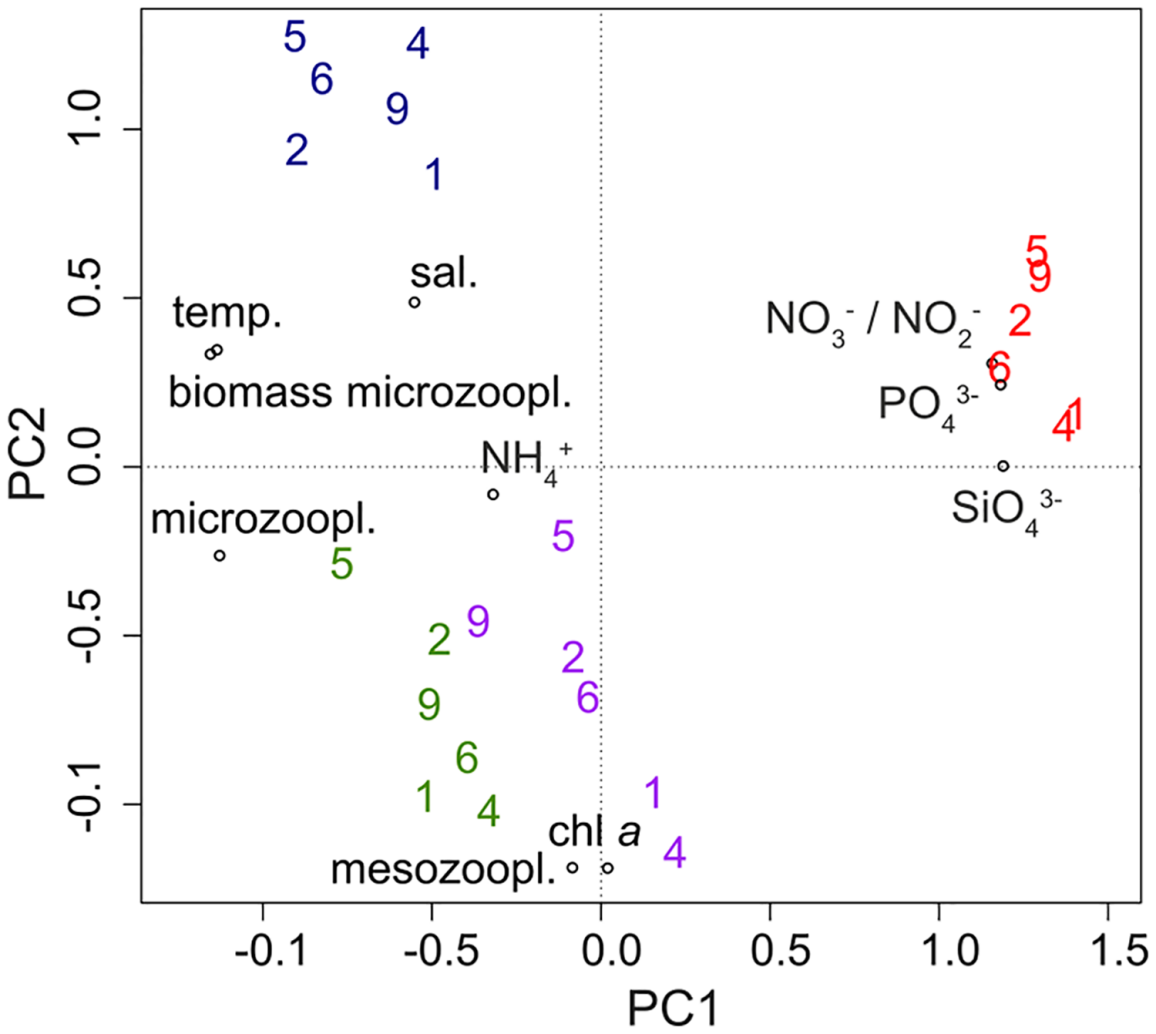
PCA of measured environmental variables and CO_2_ concentrations in the mesocosms. Mesocosms are indicated by their number. High CO_2_ (2, 4, 6) and ambient CO_2_ (1, 5, 9). The time points are indicated by color: t17 = red, t41 = purple, t65 = green and t97 = blue. Environmental variables are represented by circles, with NO_3_^-^ / NO_2_^-^ = nitrate and nitrite [μmol L^-1^], NH_4_^+^ = ammonium [μmol L^-1^], PO_4_^3-^ = phosphate [μmol L^-1^], SiO_4_^3-^ = silicate [μmol L^-1^], sal. = salinity [psu], temp. = temperature [°C], chl *a* = chlorophyll *a* [ng L^-1^], mesozoopl. = mesozooplankton [ind L^-1^], microzoopl. = microzooplankton [ind L^-1^], biomass microzoopl. = biomass microzooplankton [μg C L^-1^].

Environmental conditions differed only slightly between the investigated mesocosms (M1, M2, M4, M5, M6, M9) but showed a significant temporal development (Fig 3). The first sampling point was characterized by high nutrient (NO_3_^-^ / NO_2_^-^: 6.69 ± 0.12, PO_4_^3-^: 0.69 ± 0.00, SiO_4_^3^: 7.47 ± 0.04 [μmol L^-1^]) concentrations. Then two phytoplankton blooms followed, represented by increased chlorophyll *a* values (t41: 1363.7 ± 409.4, t65: 1858.4 ± 379.6 [ng L^-1^]), which were probably triggered by increasing water temperatures (t17: 2.19 ± 0.03, t41: 4.66 ± 0.01, t65: 8.11 ± 0.04 [°C]), resulting in the associated increase in micro- and mesozooplankton abundances (t65 microzooplankton: 6113 ± 2113, t65 mesozooplankton: 102 ± 18 [ind L^-1^]) and a simultaneous decrease in nutrients. The end of the experiment was characterized by nutrient depletion (NO_3_^-^ / NO_2_^-^ and PO_4_^3-^ concentrations were below the detection limit; SiO_4_^3^: 0.03 ± 0.05 [μmol L^-1^]), maximum water temperature (t97: 14.28 ± 0.03 [°C]), decreased mesozooplankton abundance (t97: 11 ± 2 [ind L^-1^]) and slightly increased microzooplankton biomass (t65: 33.9 ± 14.3 [μg C^-1^]). Ammonium concentration in the mesocosms stayed rather low during the experiment (t17 to t97: 0.08 ± 0.07 [μmol L^-1^]) and salinity increased slightly (t17: 29.22 ± 0.13, t97: 29.35 ± 0.11 [psu]). Values for all sampling days and mesocosms can be found in the overview paper [23].

**Fig 4:**
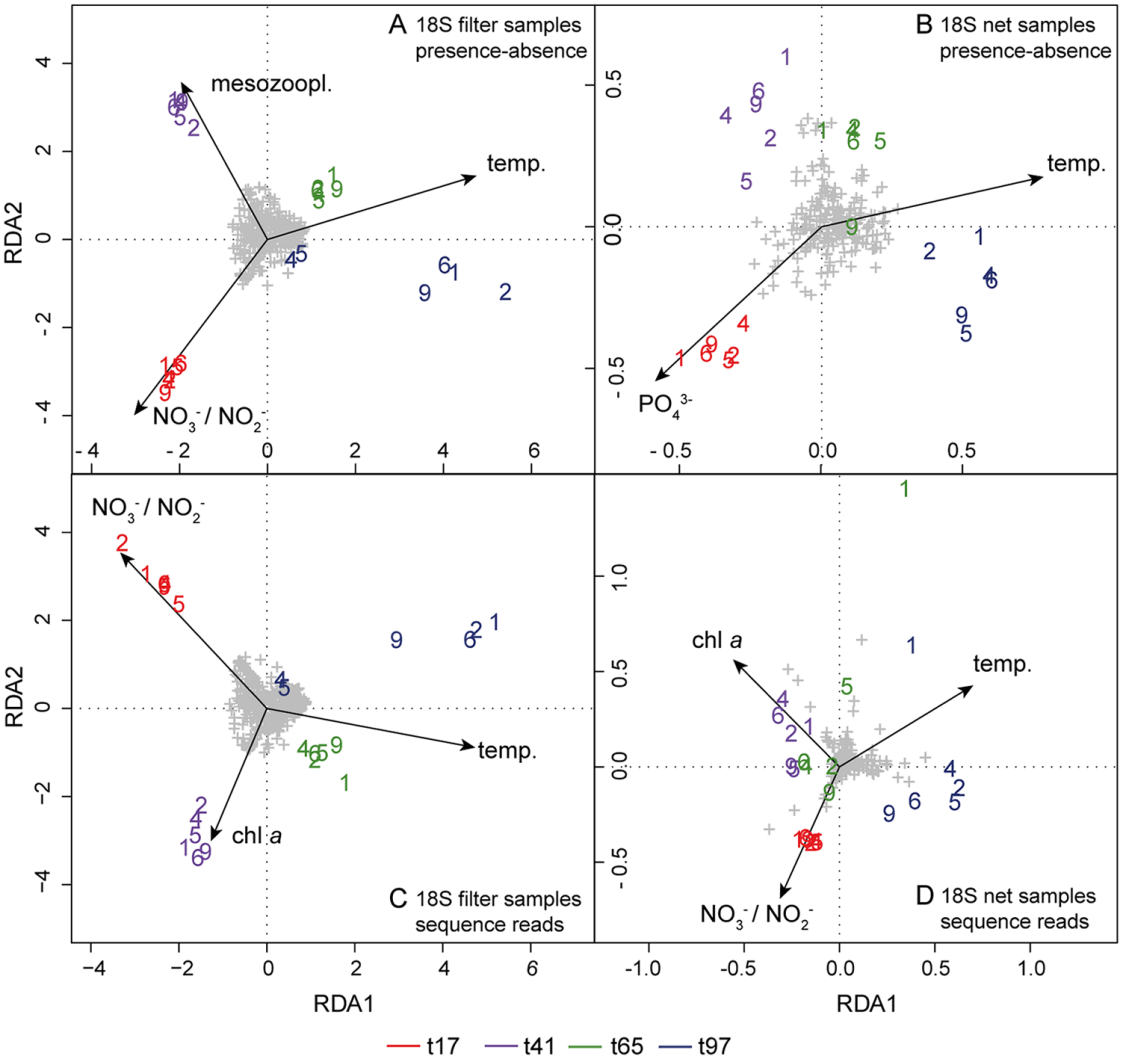
RDA of OTU compositions in dependence of ecological variables. Analysis of (A) filter samples, based on the 18S presence-absence-transformed data, (B) net samples, based on the 18S presence-absence-transformed data, (C) filter samples, based on the 18S sequence read data and (D) net samples, based on the 18S sequence read data. Mesocosms are indicated by their number. High CO_2_ (2, 4, 6) and ambient CO_2_ (1, 5, 9). OTUs are represented by grey plus signs. Time points are indicated by color: t17 = red, t41 = purple, t65 = green and t97 = blue. Explanatory variables are displayed as vectors, with NO_3_^-^ / NO_2_^-^ = nitrate and nitrite [μmol L^-1^], PO_4_^3-^ = phosphate [μmol L^-1^], temp. = temperature [°C], chl *a* = chlorophyll *a* [ng L^-1^], mesozoopl. = mesozooplankton [ind L^-1^]. Only significant ecological variables were retained for the respective RDA plot. Thus, variables shown vary for the four RDA plots.

Shifts in the OTU compositions of the 18S plankton matched the changes of environmental conditions over time as we determined based on the presence-absence-transformed and sequence read datasets (Fig 4). The OTU composition of the plankton was mainly shaped by nutrient and temperature development. After the first bloom (t41), the OTU composition (presence-absence-transformed data) of the small-sized plankton (filter samples) was additionally characterized by a high abundance of mesozooplankton. Based on the 18S sequence read data, variation among the plankton samples after the first bloom (t41) were characterized by the increased chlorophyll *a* concentration. The mesocosms were not separated by their CO_2_ treatment in the RDA ordination (ambient: M1, M5, M9; high: M2, M4, M6) indicating no CO_2_ induced significant differences in the OTU composition or number of assigned HTS reads per OTU.

### From OTU to identification: Plankton community composition and succession in terms of Taxa

**Fig 5:**
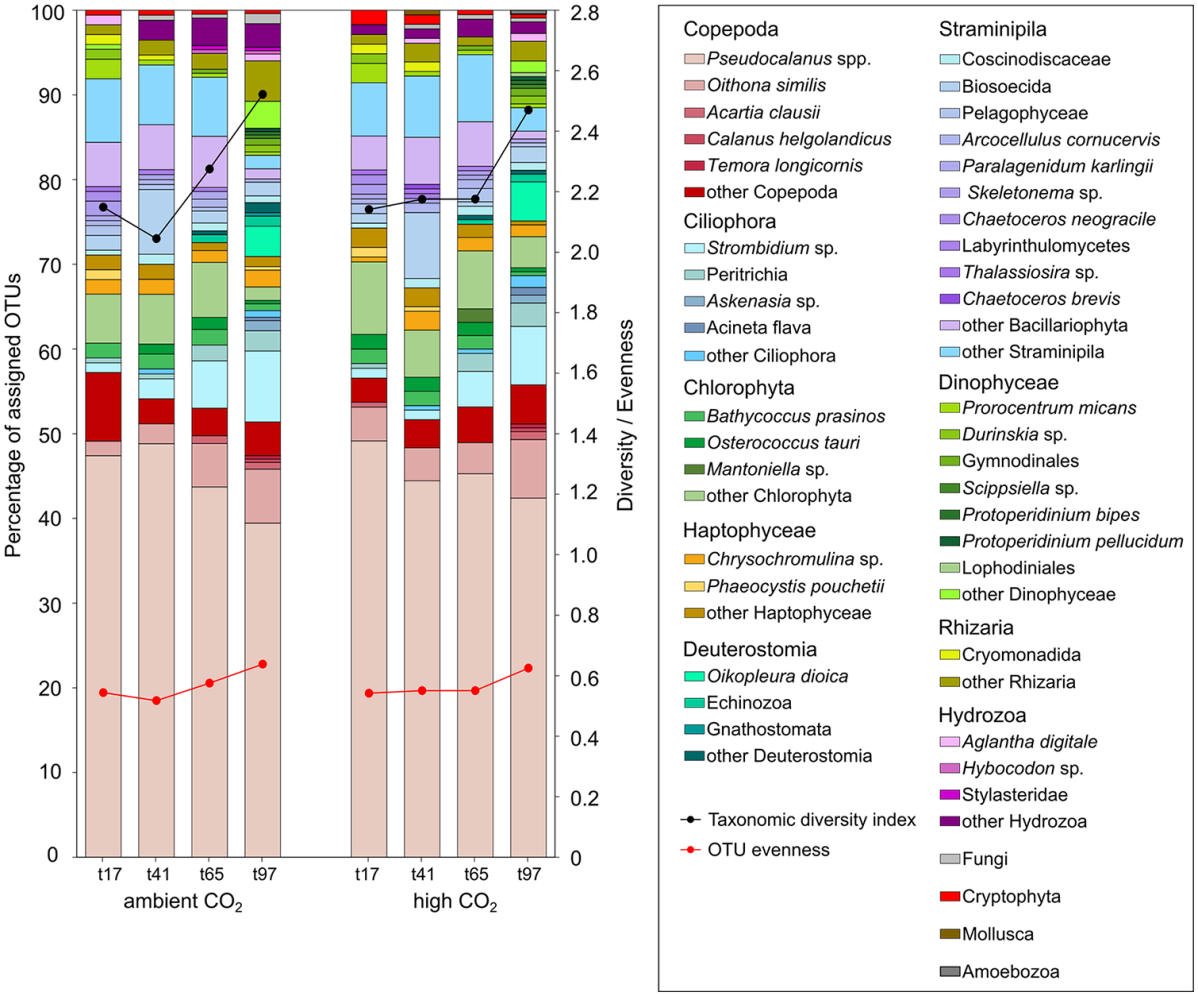
Taxonomic composition in the mesocosms. Results based on BLAST searches and MEGAN analyses of the 18S sequences. Left side ambient CO_2_ mesocosms (M1, M5, M9), right side high CO_2_ mesocosms (M2, M4, M6). Time points (t17, t41, t65, t97). The left y axis shows the percentage of assigned OTUs per taxon. The right y axis shows the taxonomic diversity index (black line) and OTU evenness (red line). Data pooled from *N = 3* replicates.

Comparing the taxonomic composition recorded with the 18S marker, in the high (M2, M4, M6) and ambient (M1, M5, M9) CO_2_ mesocosms, there were no significant differences (R^2^ = 0.02; *adonis*; *p* > 0.05). However, the plankton community composition changed significantly over time (R^2^ = 0.61; *adonis*; *p* < 0.01). The BLAST searches and MEGAN analyses of the 18S and *cox*1 OTUs resulted in the identification of 30 (± 5) and 10 (± 3) taxa per treatment and time point from different hierarchal levels, respectively. 18S OTUs assigned to various planktonic taxa within the groups Ciliophora, Dinophyceae, Amoebozoa, Fungi, Haptophyceae, Cryptophyta, Deuterostomia, Copepoda, Hydrozoa, Rhizaria, Straminipila and Chlorophyta covering all trophic levels (Fig 5). OTU evenness for the investigated 18S community was 0.57 (± 0.04), indicating the presence of dominating species in terms of biomass. Almost half of the 18S OTUs (45.1% ± 3.1) were assigned to *Pseudocalanus* spp., (later identified as *P*. *acuspes*, which was also the most abundant copepod throughout the experiment) representing its intraspecific genetic variation. Apart from copepods (54.9% ± 2.2), OTUs assigned mainly to green algae (9.3% ± 1.6), diatoms (9.2% ± 1.2) and other Straminipila (12.2% ± 3.3) at day 17 and 41. With time ciliate (t97: 13.0% ± 0.1) and dinoflagellate (t97: 6.0% ± 0.4) diversity increased. During most of the experimental period (t17, t41, t65) the recorded taxonomic diversity based on the 18S marker was quite similar among the mesocosms (Taxonomic diversity index: 2.2 ± 0.07). At the last time point (t97) taxonomic diversity increased (Taxonomic diversity index: 2.5 ± 0.03) and we detected two additional copepod species (*Calanus helgolandicus*, *Temora longicornis*), two Ciliophora (*Askenasia* sp., *Acineta flava*) and four Dinophyceae (*Scippsiella* sp., *Protoperidinum bipes*, *Protoperidinium pellucidum*, Lophodiniales) taxa as well as three other taxa (*Oikopleura dioica*, Gnathostomata, Amoebozoa) belonging to various groups.

All taxonomical groups detected in the surrounding fjord were also found in the mesocosms, although in slightly deviating diversities. Especially Bivalvia and Cirripedia were less prominent in the mesocosms.

Only 31 OTUs were obtained from the *cox*1 gene region, and MEGAN analyses resulted only in few identifiable taxa. With decreasing proportion OTUs mainly assigned to Proteobacteria (t17 to t65: 69.6% ± 10.2, t97: 37.5% ± 12.5). Note that due to the pore size of the nylon filter bacteria species smaller than 0.45 μm are not included. Beside that the *cox*1 BLAST search and MEGAN analysis delivered hits for *Pseudocalanus acuspes*, *Bathycoccus prasinos* and *Hybocodon prolifer* which supports species identification based on the 18S gene region.

Species identification based on the community barcoding was consistent with the flow cytometry investigations of the phytoplankton [23] as well as with the morphological investigations of the microzooplankton [24] and mesozooplankton (Algueró-Muñiz et al. 2017, this collection).

### *Pseudocalanus acuspes*–Species identification & intraspecific diversity

BLAST searches of the 18S sequences from the community barcoding and our reference sequences, obtained by Sanger sequencing, delivered *P*. *elongates* as the best hit (100% pairwise identity over 187 bp and 685 bp, respectively). In contrast to that, BLAST searches of our *cox*1 reference sequences (81 individuals) exclusively revealed hits for *P*. *acuspes*. Therefore, we aligned (Geneious^®^ version 7.0.4) the available NCBI 18S reference sequence of *P*. *acuspes* (GenBank: KF991205.1, 419 bp) with our own 18S reference sequences (~ 675 bp), which were proven to belong to *P*. *acuspes* by Sanger sequencing of the *cox*1 gene region of the same individuals. The alignment (89% pairwise identity over 214 bp) suggested that the sequence fragments represent different parts of the 18S gene region, which prevented an appropriate species identification using BLAST. Another investigation in the Gullmar Fjord based on species-specific PCR of 100 individuals, also resulted in the identification of *P*. *acuspes* exclusively [47]. Thus, based on these findings, we consider *P*. *acuspes* as the only present *Pseudocalanus* species throughout the experiment.

Investigations of the intraspecific genetic diversity revealed no differences between the CO_2_ treatments or sampling days, except for mesocosm two (M2). There were no CO_2_ induced differences in the OTU composition (presence-absence-transformed data, *adonis*: R^2^ = 0.008, *p* = 0.924) nor in the OTU frequencies (sequence read data, *adonis*: R^2^ = 0.010, *p* = 0.991) of the investigated *P*. *acuspes* populations. However, in M2 the OTU frequency and composition and therefore the haplotype compilation changed significantly during the experimental period (presence-absence-transformed, *adonis*: R^2^ = 0.297, *p* < 0.05; sequence read data, R^2^ = 0.308, *p* < 0.05).

## Discussion

The combination of DNA barcoding and high throughput sequencing (HTS) applied here revealed no CO_2_ induced compositional shifts in the investigated coastal plankton communities. Changes in relative taxa abundances and taxa compilations were mainly observed along the seasonal succession, which was driven by temperature and nutrient availability.

### Experimental setup

Ambient and high CO_2_ treatments remained different with respect to *p*CO_2_ throughout the whole experiment, although CO_2_ concentrations fluctuate in the mesocosms, due to outgassing and CO_2_ uptake by algae followed by additions of CO_2_ enriched water to compensate for CO_2_ losses. Starting with similar biogeochemical conditions and plankton communities, variation among mesocosm replicates increased over time (Fig 2). Initial subtle differences between the enclosures intensified over the succession. Therefore, the detection of possible CO_2_ effects became more difficult with time, because they potentially occurred at different days in the replicates. Although increasing variation among replicates is a common complication of long-term studies in natural systems, it will be a major task to resolve this problem for future studies to avoid an underestimation of the implications of increasing CO_2_.

The mesocosms included the majority of the natural plankton community; all taxa found in the surrounding fjord were also detected in the enclosures. However, the OTU composition of the mesoplankton was significantly different between the mesocosms and the fjord at the investigated time points (Fig 2). This indicates either a displacement in species succession or differences in species abundances. Some species were underrepresented in the mesocosms compared to the fjord e.g. Bivalvia and Cirripedia, which is to be expected as these meroplanktonic species typically produce their larvae later in the year, at a time when the mesocosms were long closed. Although blooms in the fjord and the mesocosms developed in parallel, they were more intense in the enclosures [23]. This suggests a similar succession, but differences in species composition and abundance among the mesocosms and the fjord.

### Community barcoding

The analyses of the community barcoding data resulted in a total number of 771 OTUs with an identity cutoff of 99%. This indicated a high sequencing depth and thereby a high coverage of the species (and intraspecific genetic variation) diversity present in the mesocosms and in the surrounding fjord. The plankton communities in the three ambient (M1, M5, M9) and high CO_2_ (M2, M4, M6) mesocosms were represented by 375 ± 66 OTUs per time point. During the MEGAN analyses, only around one half of the OTUs (198 ± 33) could be assigned to reference sequences. The other half could not be assigned, probably because corresponding 18S reference sequences are missing in the database yet. Furthermore, multiple OTUs were assigned to the same taxa, since some OTUs represent haplotypes of the same species. Overall, based on the 18S and *cox*1 sequences we could identify 19 species, 9 genera, 16 higher taxonomical groups i.e. 44 different taxa. The success of studies such as the present depends very much on the quality of the available molecular databases. For a steady improvement of those databases good cooperation between taxonomists and molecular biologists is therefore crucial.

Despite the high taxonomic resolution based on OTUs, metabarcoding data cannot be used to make predictions about species abundances and/or biomasses on-site, due to preferential annealing of universal primers in some species over others during PCRs and HTS [48]. Therefore, relative abundances and diversity indices, based on HTS reads and the number of OTUs assigned to certain taxa, respectively, are not directly comparable to those determined using count abundance data. However, comparisons among the enclosures and time points are feasible because amplification and sequence success between should be constant.

Based on the number of assigned OTUs to a certain taxon, predictions about the intraspecific diversity is only possible to a limited extent. For investigations of the intraspecific diversity a sufficient sample size is crucial, because up to a certain point the observed intraspecific variation is positively correlated with the number of investigated individuals. Therefore, the percentages of assigned OTUs shown in Fig 5 do not reflect the actual level of intraspecific variation, since it is unlikely that a representative sample size could be reached for all taxa detected with the community barcoding. *P*. *acuspes*, however, was the most abundant copepod (based on mesozooplankton counts, Algueró-Muñiz et al. 2017, this collection) throughout the experiment and with 1,351 (± 561) assigned sequence reads per sample also the best covered taxa in the HTS of the 18S gene region. We observed no effect of elevated CO_2_ on the genetic intraspecific diversity of the investigated *P*. *acuspes* populations. Furthermore, in all investigated mesocosms (M1, M4, M5, M6, M9) except M2 the OTU, and thus the haplotype compilations of the *P*. *acuspes* populations, were similar at the sampling days 17, 41, 65, and 97. The temporal change in the OTU composition of the *P*. *acuspes* population of M2 was probably due to a decline in the number of detected OTUs (~ 40 less) on the filter sample on day 97. Since the intraspecific diversity decrease was only detected in one of the three replicates and we further cannot exclude a potential methodological flaw this finding should not be overinterpreted.

Unexpectedly, a relatively high number of 18S OTUs (115) assigned to *P*. *acuspes*, which might indicate that several cryptic lineages co-occur in this species complex. However, such a high level of intraspecific variation is unusual for the rather conserved 18S gene region. Since other processes such as sequencing errors might explain an increased number of assigned OTUs, one should interpret this finding with caution. Additionally, we did not observe such a high intraspecific variation using *cox*1 reference sequences (81 individuals, 7 haplotypes). Nevertheless, the actual genetic variation might have been underestimated due to the relatively low number of investigated individuals compared to the community barcoding data set. The HTS of the *cox*1 gene region delivered only one OTU assigned to *P*. *acuspes* (containing 14 DNA sequences), i.e. not a representative sample of the population. Further investigations are necessary to generate a sound statement about the actual intraspecific genetic diversity of the *P*. *acuspes* species complex.

The OTU distribution among the filter and net samples was quite different. A higher proportion of the OTUs originated from filter samples. However, this is not surprising since species diversity is in general higher in small organisms (here microplankton) compared to large ones (here mesoplankton) and taxa larger than 1 mm were excluded (which were very rare). Around 50% of the OTUs corresponded to both size fractions (filter and net samples), because eggs, larvae and nauplii of mesozooplankton as well as smaller cells of large diatoms (e.g. Coscinodiscaceae) are also captured on the filter samples. Furthermore, pico-, nano- and microplankton were ingested by larger planktonic taxa or just attached to them and will be detected along with the grazer/predator.

### CO_2_ effects on the plankton community composition and food web interactions

The composition of plankton communities investigated in this mesocosm study was not affected by increased CO_2_ levels. At the analysed time points (t17, t41, t65, t97) no significant differences between the CO_2_ treatments could be observed. Yet, there were significant compositional changes over time (Fig 2), which were primarily induced by changing temperature and decreasing nutrient availability over the course of the succession (Fig 4). Additionally, at day 41, the 18S OTU composition (presence-absence-transformed data) of the small-sized plankton communities (pico-, nano- and microplankton) also seemed to be driven by mesozooplankton abundances, i.e. rather by top-down effects from predators than by bottom-up effects from food sources (Fig 4A and 4B). Löder et al. (2011) found copepods to be less important phytoplankton grazers, but important top-down regulators of microzooplankton, especially with decreasing quality of phytoplankton food due to nutrient limitation during blooms [49]. Furthermore, many copepods are size-selective feeders, preferring a size class between 10–10^2^ μm [50]. During the bloom events the phytoplankton community was mainly made up by picoeukaryotes (< 2 μm) and *Coscinodiscus* sp. (> 200 μm), a giant diatom. Therefore, the vast majority of the phytoplankton present did not represent a suitable food source for copepods, due to the unfavorable cell size. Thus, copepods probably intensively grazed on microzooplankton organisms [24], most likely ciliates and dinoflagellates. This is corroborated by the 18S mesoplankton OTU composition (presence-absence-transformed data) obviously not being related to phytoplankton densities (chlorophyll *a*) in the RDA analysis (Fig 4A and 4B). On day 97 towards the end of the experiment more OTUs assigned to ciliate and dinoflagellate species (Fig 5), after the top-down control by mesoplankton subsided due to decreasing copepod abundances. Considering the amount of assigned HTS reads per OTU in the RDA the predator-prey food web interaction was probably masked, because the high numbers of sequence reads, which correspond to OTUs representing the main contributing taxa of the blooms, (Chlorophyta, Rhizaria and Straminipila) carried more weight in the analysis then the actual composition of the OTUs. Therefore, in the RDA, variation between plankton samples at day 41 were rather explained by the chlorophyll *a* concentration than by the abundance of mesozooplankton. The introduced herring larvae potentially controlled both micro- and mesozooplankton abundances. In their early life stages from day ~ 64 to 74 they were probably feeding first on small-sized microzooplankton e.g. ciliates and subsequently on larger microzooplankton organisms and nauplii stages of mesozooplankton taxa. Towards the end of the experiment they most likely started to feed on adult mesozooplankton organisms. As *P*. *acuspes* was the dominant copepod species it was probably mostly consumed by the herring larvae. Decreasing densities of *P*. *acuspes* towards the end of the experiment, thereby potentially facilitated the development of other copepod species (Fig 5) due to reduced competition.

As part of this mesocosm experiment other studies found temporal effects of high CO_2_ on planktonic taxa. Dinoflagellates were more abundant in the high CO_2_ mesocosms towards the end of the experimental period [24], and the phytoplankton community structure was significantly different between the CO_2_ treatments during the second bloom period, with a higher abundance of picophytoplankton [23]. Lischka et al. (2015) also observed temporal effects of elevated CO_2_ on a microzooplankton community in Tvärminne / Storfjärden, whereby *inter alia* a shift towards smaller taxa with increasing CO_2_ was detected [51]. The second bloom was fueled by recycled nutrients, thus resources for plankton growth needed to by delivered by the food web, thereby the limitation of inorganic nutrients seemed to indirectly enhance CO_2_ effects on planktonic organisms. The higher Proteobacteria diversity (number of assigned OTUs based on the *cox*1 gene region) in the period after the blooms (t41, t65) may also suggest a rather bottom-up driven food web. A similar pattern was found by Fierer et al. (2007) who showed a positive correlation between the abundance of β-Proteobacteria and C mineralization rates [52]. After decomposition and remineralization of the particulate organic matter, Proteobacteria diversity decreased towards day 97. Dependencies between nutrient availability and impacts of elevated CO_2_ on marine organisms were also demonstrated in previous studies [53–56]. Therefore, future studies investigating the impacts of OA on marine biota should focus on nutrient limited systems.

### Study hypothesis

Based on the results of this study our hypothesis that DNA barcoding in combination with HTS can unravel previously hidden (based on morphological analyses) CO_2_ sensitivities of plankton communities cannot be confirmed. We focused on the investigation of potential long-term effects of CO_2_ on planktonic taxa compilations, because the number of samples for the HTS was limited (financial constrains). Thereby, samples from the distinct bloom events were not considered for the community barcoding. Thus, we did not assess whether the CO_2_ induced increase in the abundance of picoeukaryotic phytoplankton [23], which was observed during the second bloom, could have been detected with community barcoding. Although our genetic approach allowed a detailed investigation of the majority of the planktonic communities (down to haplotype level), the technique is not appropriate to detect slight changes in species abundances and stage specific effects, as it has been observed for some dinoflagellate [24] and copepod taxa (Algueró-Muñiz et al. 2017, this collection), respectively. However, community barcoding data delivers additional information about the genetic diversity of a community and the presence of potentially cryptic or even unknown species. Thus, for future studies we suggest to use community barcoding as applied here, not as a standalone technique but as a tool to complement classical morphological investigations.

## Conclusions

Based on the community barcoding data elevated CO_2_ had no significant effect on the relative abundance or compilation of coastal planktonic taxa during a winter-to-summer succession in the mesocosms investigated in this field experiment. The resilience of coastal plankton communities (bacteria, phytoplankton, micro- and mesozooplankton) towards future OA conditions was also demonstrated in earlier large scale mesocosm field and indoor studies [9–13, 57, 58]. In agreement with those results, we assume that low sensitivities for high CO_2_ conditions are common for coastal plankton communities which are exposed to pronounced natural fluctuations in seawater pH.

## Supporting information

S1 Table Final OTU table based on the 18S gene region containing the HTS reads. Columns A—E contain information about the sampling day, sampling method, sampled mesocosms and CO_2_ treatment. Columns F—P contain information about environmental variables. Columns Q—ACB contain OTU information. This data was used for adonis, PCA (Fig 3), nMDS (Fig 2) and RDA (Fig 4) analysis.
(TXT)

S2 Table Final OTU table based on the *cox*1 gene region containing the HTS reads. Columns A—E contain information about the sampling day, sampling method, sampled mesocosms and CO_2_ treatment. Columns F—AN contain OTU information. This data was used for adonis analysis.
(TXT)

S3 Table Steps of the bioinformatics pipeline. Including the programs used, decreasing sequence reads, and OTU numbers.
(PDF)
