# Supplementary material for: Community barcoding reveals little effect of ocean acidification on the composition of coastal plankton communities: Evidence from a long-term mesocosm study in the Gullmar Fjord, Skagerrak
Source: PLoS One. 2017 Apr 26;12(4):e0175808. doi: 10.1371/journal.pone.0175808 (PMC5405915; doi:10.1371/journal.pone.0175808)
Supplement: S3 Table — Including the programs used, decreasing sequence reads, and OTU numbers. (PDF) [file pone.0175808.s003.pdf]

**Pipeline step**	**Program**	**File**	**Reads**	**OTUs**
Raw sequence data		forward<br>reverse	4,987,876<br>4,987,876	
1. Illumina adapter trimming and filtering	Trimmomatic	forward<br>reverse	1,630,884<br>1,630,884	
2. Quality filtering	FastQFS	forward<br>reverse	1,630,807<br>1,630,807	
3. De-multiplexing<br>4. Barcode and primer trimming<br>5. Reorient reads 5' – 3'		18S<br>*cox*1	653,789<br>3,417	
6. Similarity clustering	USEARCH v7	18S<br>*cox*1		7,734<br>96
7. Re-assemble	Geneious® 7.0.4	18S<br>*cox*1		1,037<br>39
8. Alignment	Geneious® 7.0.4	18S<br>*cox*1		851<br>36
9. Mapping	USEARCH v7	18S<br>*cox*1		826<br>35
10. OTU abundance table	Microsoft Excel (2016)	18S<br>*cox*1	536,609<br>2,638	740<br>31
